# Development of a Novel Six-miRNA-Based Model to Predict Overall Survival Among Colon Adenocarcinoma Patients

**DOI:** 10.3389/fonc.2020.00026

**Published:** 2020-02-21

**Authors:** Zhenxiang Rong, Yi Rong, Yingru Li, Lei Zhang, Jingwen Peng, Baojia Zou, Nan Zhou, Zihao Pan

**Affiliations:** ^1^Department of General Surgery, New Rongqi Hospital, Foshan, China; ^2^Zhongshan School of Medicine, Sun Yat-sen University, Guangzhou, China; ^3^Department of Gastroenterology, Hernia and Abdominal Wall Surgery, The Sixth Affiliated Hospital, Sun Yat-sen University, Guangzhou, China; ^4^Biliary-Pancreatic Surgery, The Third Affiliated Hospital, Sun Yat-sen University, Guangzhou, China; ^5^Guangdong Provincial Key Laboratory of Malignant Tumor Epigenetics and Gene Regulation, Sun Yat-sen Memorial Hospital, Sun Yat-sen University, Guangzhou, China; ^6^Department of Hepatobiliary Surgery, The Fifth Affiliated Hospital of Sun Yat-sen University, Zhuhai, China

**Keywords:** colon adenocarcinoma, microRNA, TCGA, nomogram, model

## Abstract

**Introduction:** Colon carcinoma is a common malignant tumor worldwide. Accurately predicting prognosis of colon adenocarcinoma (CA) patients may facilitate clinical individual decision-making. Many studies have reported that microRNAs (miRNAs) were associated with prognosis for patients with colon carcinoma. This study aimed to identify the prognosis-related miRNAs for predicting the overall survival (OS) of CA patients.

**Methods:** Firstly, we analyzed the CA datasets from the Cancer Genome Atlas (TCGA), and looked for the prognosis-related miRNAs. Then, we developed a novel prediction model based on these miRNAs and the clinical characteristics. Time-dependent receiver operating characteristics (ROC) curves and calibration plots were used to evaluate the discrimination and accuracy of the signature and model. Finally, cell function assays and bioinformatics analyses were performed to evaluate the role of these selected miRNAs in modulating biological process in CA.

**Results:** Six prognosis-related miRNAs were included in the miRNA-based signature, and it could effectively distinguish low-risk patients and high-risk patients. Furthermore, we established a prognostic model incorporating the six-miRNA-based signature and clinical characteristics. Areas under curves (AUCs) indicated that the six-miRNA-based model has a better predictive ability than TNM stage (AUC: 0.805 vs. 0.694). The calibration plots suggested close agreement between model predictions and actual observations. GO analysis showed that the target genes of these miRNAs are mainly involved in enrichment in protein binding and regulation of transcript and cytosol. KEGG pathway enrichment analysis indicated that these genes were mainly enriched in PI3K-Akt signaling pathway. Finally, we found that the five miRNAs except miR-152 were upregulated in tumor tissues and CA cells. The functional experiments revealed that miR-1245a, miR-3682, miR-33b, and miR-5683 promoted the migratory abilities and proliferation of CA cell, whereas miR-152 showed opposite effects. However, miR-4444-2 did not influence the migratory ability and proliferation of CA cell.

**Conclusions:** In conclusion, we developed a novel six-miRNA-based model to predict 5-year survival probabilities for CA patients. This model has the potential to facilitate individualized treatment decisions.

## Introduction

Colon carcinoma is a common malignant tumor worldwide, which is the fourth leading cause of cancer-related deaths (1). Among all histological types, colon adenocarcinoma (CA) is the most common one with a poor prognosis (2). Although the therapeutic strategies for CA have been greatly improved, the 5-year overall survival (OS) rate remained poor. The biomarkers for CA, such as CA199 and CEA, lack good specificity and sensitivity for recognizing all primary and recurrent lesions for all patients. In addition, TNM stage, as a common tool for the prognostic assessment mainly on the basis of clinical characteristics, could not display the biological heterogeneity of CA. In clinical practice, accurately predicting OS for CA patients may facilitate clinical individual decision-making. Therefore, accurate prediction tools integrating the clinical features and pathological and molecular findings are much desired.

MicroRNA (miRNA), a small non-coding RNA with 18–25 nucleotides, can regulate gene expression in various carcinomas (3). According to current reports, several of these miRNAs are important to CA patients because of their usefulness in making diagnoses, evaluation of treatment responses, as well as the prediction of prognosis (4–7). However, different miRNA platforms were explored in these previous studies with limited patient samples, suggesting that they lack normalized standard of miRNAs. A series of novel prediction models based on miRNAs were developed in several cancers, including breast cancer, esophageal squamous cell cancer, and acute myeloid leukemia (8–10). These models indicated that miRNAs play important and valid roles in predicting the prognosis of cancer.

In this study, we mined the CA datasets from the Cancer Genome Atlas (TCGA) and screened the prognosis-related miRNAs. Then, we developed and validated a novel prediction model based on these prognosis-related miRNAs and clinical characteristics. Finally, bioinformatics analyses and cell function assays were performed to evaluate the role of these selected miRNAs in modulating biological process in CA.

## Materials and Methods

### Patients and Design

In the present study, we acquired the counts of CA miRNA expression profiles from TCGA data portal in May 2019, and available miRNAs were 1,881. The eligibility criteria used to screen the included patients were as follows: (1) patients with certain TNM stage; (2) histologically confirmed CA; and (3) OS time was more than 3 months. Finally, CA patients with clinical characteristics including age, sex, T stage, N stage, and TNM stage were analyzed in the present study and defined as training cohort. Additionally, half of the patients were assigned as the validation cohort based on a computer-generated allocation sequence randomly.

### Risk Score Formula Generation and Development of miRNA-Based Prognostic Model

We normalized the miRNA expression profiles through R/Bioconductor package of edger. All miRNAs with FDR < 0.05 and |log2FC| ≥ 2 were defined as differently expressed miRNAs (DEMs). We used Cox proportional hazards regression analysis to screen for miRNAs. Significant miRNAs were included in the multivariate Cox proportional hazards regression model. The coefficients of significant miRNAs from multivariate Cox analysis was used to build the risk score formula: Risk score = sum of coefficients × miRNA expression level.

Next, the CA patients were classified into low-risk group and high-risk-group using the ROC curve with the optimal cutoff risk score. Furthermore, we developed a prognostic nomogram that incorporated clinical characteristics and miRNA-based risk score with the COX regression model.

### Evaluation of miRNA-Based Signature and Novel Prediction Model

To test whether miRNA-based signature was related to OS independent of TNM stage, stratified analysis was performed. Additionally, we used time-dependent receiver operating characteristic (ROC) curves and calibration plots to assess the prognostic models' discriminative ability and accuracy, respectively. Calibration plots were used to measure the agreement between the actual and predicted probabilities. The predictive ability of miRNA-based prognostic model was compared with six-miRNA-based signature and TNM stage using ROC curves.

### Enrichment Analysis of miRNAs' Target Genes

Candidate target genes of prognostic miRNAs were obtained from miRDB, TargetScan, and miTarBase. Overlapping genes in the three online resources are selected as miRNAs' target genes. The interaction network of interactions between miRNAs and target genes was visualized using Cytoscape (11). At last, GO (Gene Ontology) and KEGG (Kyoto Encyclopedia of Genes and Genomes) pathway enrichment analyses were carried out using DAVID 6.8 (Database for Annotation, Visualization and Integrated Discovery).

### Total RNA Extraction and Quantitative Real-Time PCR

We extracted RNA sample with RNAiso Plus (TaKaRa, Japan) according to the manufacturer's instructions, and the measure of concentration and purity was completed by NanoDrop 2000 (Thermo Scientific, Wilmington, DE, USA). All cDNA was generated using PrimeScript RT reagent Kit (TaKaRa, Japan). Quantitative real-time PCR (qRT-PCR) for miRNAs was performed on a LightCycler® 96 System (Roche, Switzerland).

### Cell Culture

CA cell lines (DLD-1 and SW480) and normal colon mucosal epithelial cell line (NCM460) were cultured in DMEM medium with 10% fetal bovine plasma at 37°C in a humidified atmosphere with 5% CO_2_, and they were transfected with miRNA inhibitors for 48 h before cell function experiments.

### Transwell Migration Assays

The transwell assays were used to evaluate the migration of DLD-1 and SW480 cells using transwell chambers. In test, 2 × 10^5^ cells in serum-free medium were added into the upper chambers. DMEM medium with 10% fetal bovine serum was added to the lower chambers. After incubation for 24 h, the CA cells migrated into the lower chambers were fixed in 4% paraformaldehyde and strained with a crystal violet staining solution. Random fields were digitally imaged and counted.

### Colony Formation Assays

We used the colony-forming assays to evaluate clonogenic ability of transfected DLD-1 and SW480 cells. Cells were seeded into 6-well plates (1,000/well) and incubated for about 15 days. The visible colonies were counted after staining with crystal violet.

### Statistics

Descriptive analyses of baseline clinicopathological features were conducted. Continuous variables were reported using the mean and standard deviation (SD), and categorical variables were reported as percentages. In developing the nomogram for predicting the 5-year OS probability, we used univariate and multivariate Cox proportional hazards regression model to screen for predictors in the training cohort. OS was defined as the interval from surgery to the date of death. Then, the nomogram was developed based on the significant predictors. The nomogram's discriminative ability and accuracy were assessed by time-dependent receiver operating curve (ROC) analysis and calibration plots, respectively. Calibration plots were used to measure the agreement between the actual and predicted probabilities. *P* < 0.05 was considered statistically significant. We draw the volcano plot using the “ggplot2” package of R software. Data analyses were performed using Stata version 13.1 (StataCorp, College Station, TX), and the nomogram was developed using R (version 3.2.4; R Foundation for Statistical Computing, Vienna, Austria).

## Results

By the eligibility criteria, 321 CA patients from TCGA were included in training cohort. For further analyses, 161 patients were divided randomly into the validation cohort. In Table 1, there were the baseline features of patients in the training cohort and the validation cohort. The 5-year OS rate of the total patients was 74.3%.

**Table 1:** Baseline characteristics of CA patients from TCGA.

	**Training cohort (*****n*** **=** **321)**		**Validation cohort (*****n*** **=** **161)**		
**Variables**	***N***	**%**	***N***	**%**	***P*-value**
Age (years)	66.59 ± 12.43		66.53 ± 12.10		0.963
Age					0.820
≤ 69	173	53.89	85	52.80	
>69	148	46.11	76	47.20	
Sex					0.871
Male	170	52.96	84	52.17	
Female	151	47.04	77	47.83	
T stage					0.993
T1	7	2.18	4	2.48	
T2	58	18.07	29	18.01	
T3	222	69.16	112	69.57	
T4	34	10.59	16	9.94	
N stage					0.816
N0	193	60.12	100	62.11	
N1	74	23.05	33	20.50	
N2	54	16.82	28	17.39	
TNM stage					0.975
I	59	18.38	31	19.25	
II	129	40.19	66	40.99	
III	88	27.41	41	25.47	
IV	45	14.02	23	14.29	

### Screen the Prognostic miRNAs Related to OS

**Figure 1:**
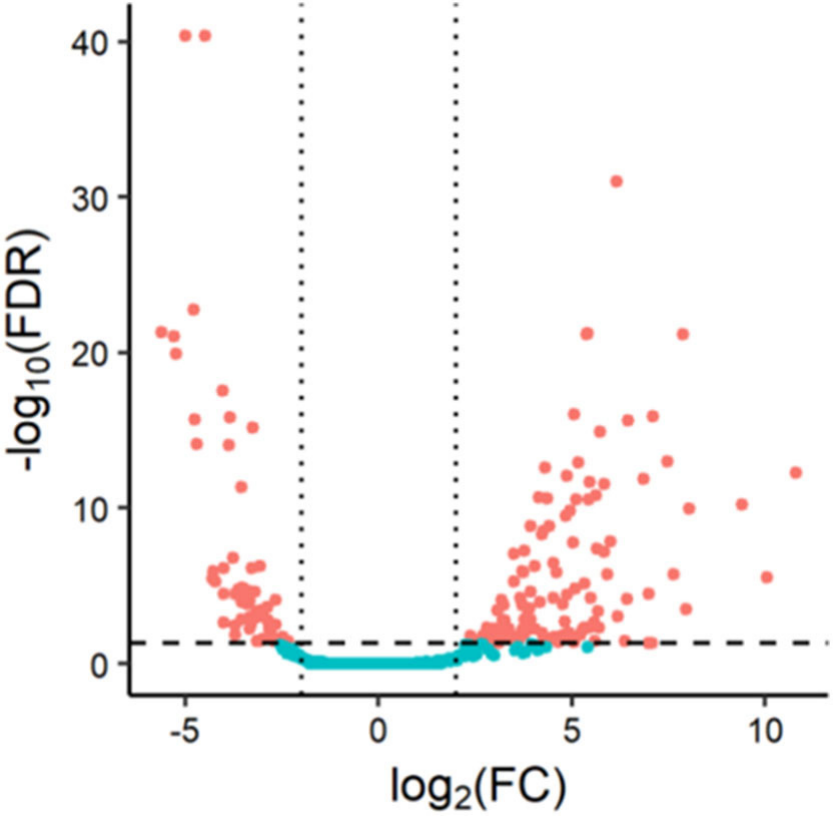
Volcano plot of 1,881 miRNAs in colon adenocarcinoma patients. Red color represents dysregulated expression.

We obtained miRNA sequencing data of samples from TCGA database, including CA tissue samples and normal tissue samples. Next, through the R/Bioconductor package of edgeR, 190 DEMs with false discovery rate (FDR) < 0.05 and |log2fold change (log2FC)| ≥ 2 were suggested to be significant for further analyses. The volcano plot of all miRNAs was presented using the “ggplot2” package of R software (Figure 1). To screen the prognostic miRNAs, 190 DEMs were performed in univariate COX analysis. Then, 42 miRNAs that were significant in the univariate COX analysis were entered into multivariate COX analysis. As a result, six DEMs (hsa-miR-1245a, hsa-miR-3682, hsa-miR-4444-2, hsa-miR-5683, hsa-miR-33b, and hsa-miR-152) were confirmed as independent prognosis factors of CA patients in the training cohort (Table 2).

**Table 2:** Six prognostic miRNAs significantly associated with OS in training cohort.

**Name**	**Coefficient**	**Type**	**HR**	**95% CI**	***P*-value**
miR-1245a	0.0903387	Risky	1.095	1.006–1.191	0.036
miR-3682	0.1288864	Risky	1.138	1.012–1.279	0.031
miR-4444-2	0.1769175	Risky	1.194	1.008–1.413	0.040
miR-5683	0.0103112	Risky	1.010	1.006–1.0145	<0.001
miR-33b	0.0223187	Risky	1.023	1.004–1.042	0.019
miR-152	−0.0013202	Protective	0.998	0.997–0.999	0.047

*OS, overall survival; HR, hazard ratio; CI, confidence interval*.

### Construction of Risk Score Signature and Six-miRNA-Based Prognostic Model

**Figure 2:**
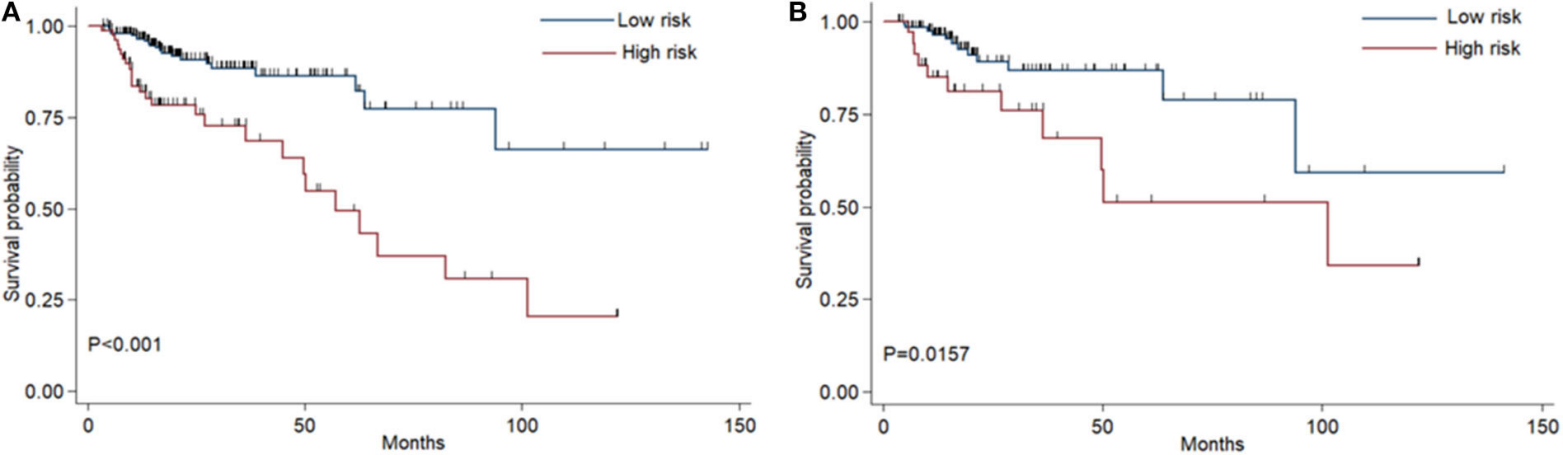
Kaplan–Meier curves of overall survival for colon adenocarcinoma patients based on the six-miRNA signature in the training cohort **(A)** and validation cohort **(B)**.

**Figure 3:**
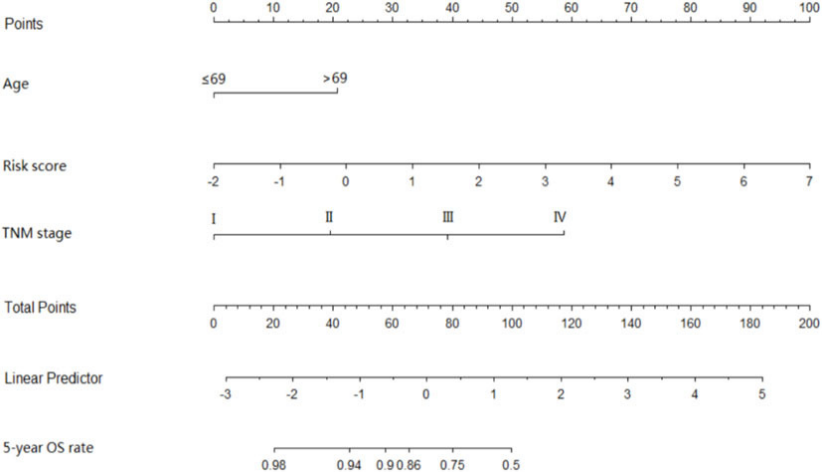
Six-miRNAs-based prognostic model to predict 5-year overall survival in colon adenocarcinoma. To use this nomogram, we use the “point” scale to estimate the points for each variable by drawing a vertical line. For example, if a 60-year-old patient (0 points) with TNM II (18 point) has miRNA risk score 0 (21 points), the total point of this man is 39. Then, we can use the “Total points” scale to estimate the corresponding 5-year overall survival of this patient.

The coefficients of significant miRNAs from multivariate Cox analysis were used to build the risk score formula: Risk score = sum of coefficients × miRNAs expression level. Therefore, the risk score signature was calculated using the formula below: risk score = (0.0903387 × expression_miR−1245a_) + (0.1288864 × expression_miR−3682_) + (0.1769175 × expression_miR−4444−2_) + (0.0103112 × expression_miR−5683_) + (0.0223187 × expression_miR−33b_) – (0.0013202 × expression_miR−152_). Moreover, in training cohort, the risk score of each patient was calculated. Then, we used ROC curve to determine the optimal cutoff risk score for stratifying the patients into risk groups. When the Youden index was highest, the risk score is 1.58 with a sensitivity and specificity of 56.25% and 80.22%, respectively. Therefore, the patients were divided into a high-risk group (*n* = 81) and a low-risk group (*n* = 240) in the training cohort. With the cutoff value, the patients in the validation cohort were also divided into a high-risk group (*n* = 35) and a low-risk group (*n* = 126). The predictive value of six-miRNA-based signature in OS was detectable through Kaplan–Meier curve of two cohorts as shown in Figure 2. Patients with high risk have a poorer survival than the low-risk group in training cohort (*P* < 0.001) and validation cohort (*P* = 0.0157). Next, to develop a six-miRNA-based prognosis model, we used univariate and multivariate COX analysis to identify risk factors. Finally, the six-miRNA-based signature (HR = 1.65, 95% CI 1.36–1.99, *P* < 0.001), age (>69 vs. ≤69, HR = 2.58, 95% CI 1.40–4.76, *P* = 0.002), and TNM stage (III vs. I, HR = 3.83, 95% CI 1.09–13.45, *P* = 0.036; IV vs. I, HR = 8.22, 95% CI 2.37–28.53, *P* = 0.001) were confirmed as independent prognosis factors of OS (Table 3). As a result, a novel six-miRNA-based prognostic model to predict the 5-year OS rate was developed based on the above three variables (Figure 3). In the nomogram, it showed that six-miRNA-based signature and TNM stage were the largest contribution to 5-year OS of CA patients. To use this nomogram, we use the “point” scale to estimate the points for each variable by drawing a vertical line. Then, the “Total points” scale was used to estimate the corresponding 5-year OS of this patient.

**Table 3:** Univariate and multivariate COX proportional hazards regression analyses in training cohort.

**Variables**	**Univariate analysis**			**Multivariate analysis**		
	**HR**	**95% CI**	***P*-value**	**HR**	**95% CI**	***P*-value**
Age						
≤ 69	1			1		
>69	1.96	(1.09, 3.52)	0.024	2.58	(1.40, 4.76)	0.002
Sex						
Female	1					
Male	1.75	(0.97, 3.17)	0.065			
T Stage						
T1+T2	1					
T3	2.29	(0.69, 7.59)	0.175			
T4	10.4	(2.93, 36.93)	<0.001			
N Stage						
N0	1					
N1	2.84	(1.38, 5.86)	0.004			
N2	5.07	(2.58, 9.98)	<0.001			
TNM Stage						
I	1			1		
II	1.22	(0.34, 4.40)	0.752	1.18	(0.33, 4.25)	0.803
III	2.89	(0.83, 10.05)	0.095	3.83	(1.09, 13.45)	0.036
IV	7.53	(2.20, 25.81)	0.001	8.22	(2.37, 28.53)	0.001
Six-miRNA-based-signature	1.74	(1.44, 2.11)	<0.001	1.65	(1.36, 1.99)	<0.001

*HR, hazard ratio; CI, confidence interval*.

### Assessment of the Six-miRNA-Based Signature and Novel Six-miRNA-Based Prognostic Model

**Figure 4:**
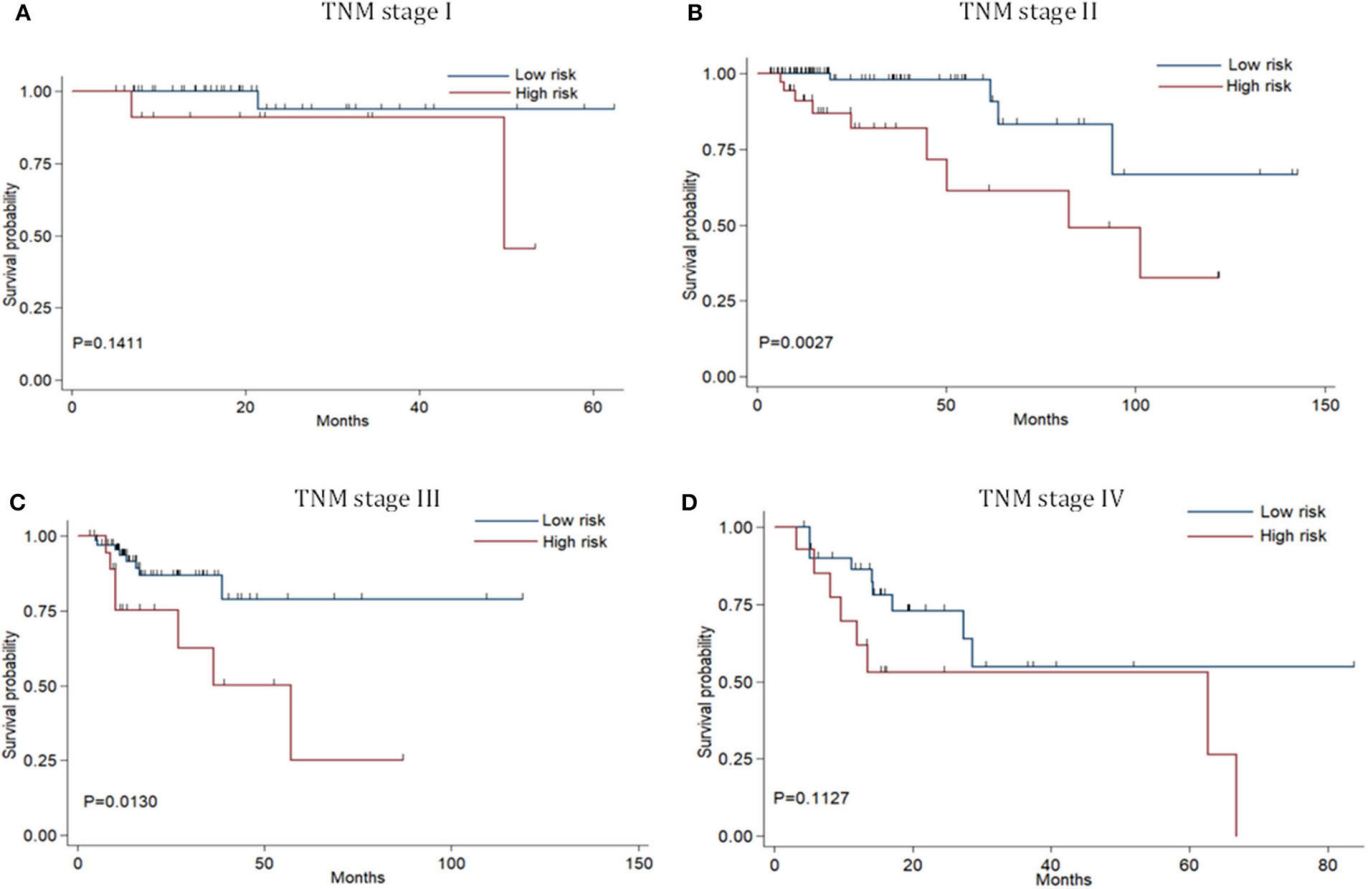
Stratified analysis of six-miRNA-based signature for colon adenocarcinoma patients in TNM stage. Kaplan-Meier curves of over survival for CA patients in TNM stage I **(A)**,TNM stage II **(B)**,TNM stage III **(C)**, and TNM stage IV **(D)**.

**Figure 5:**
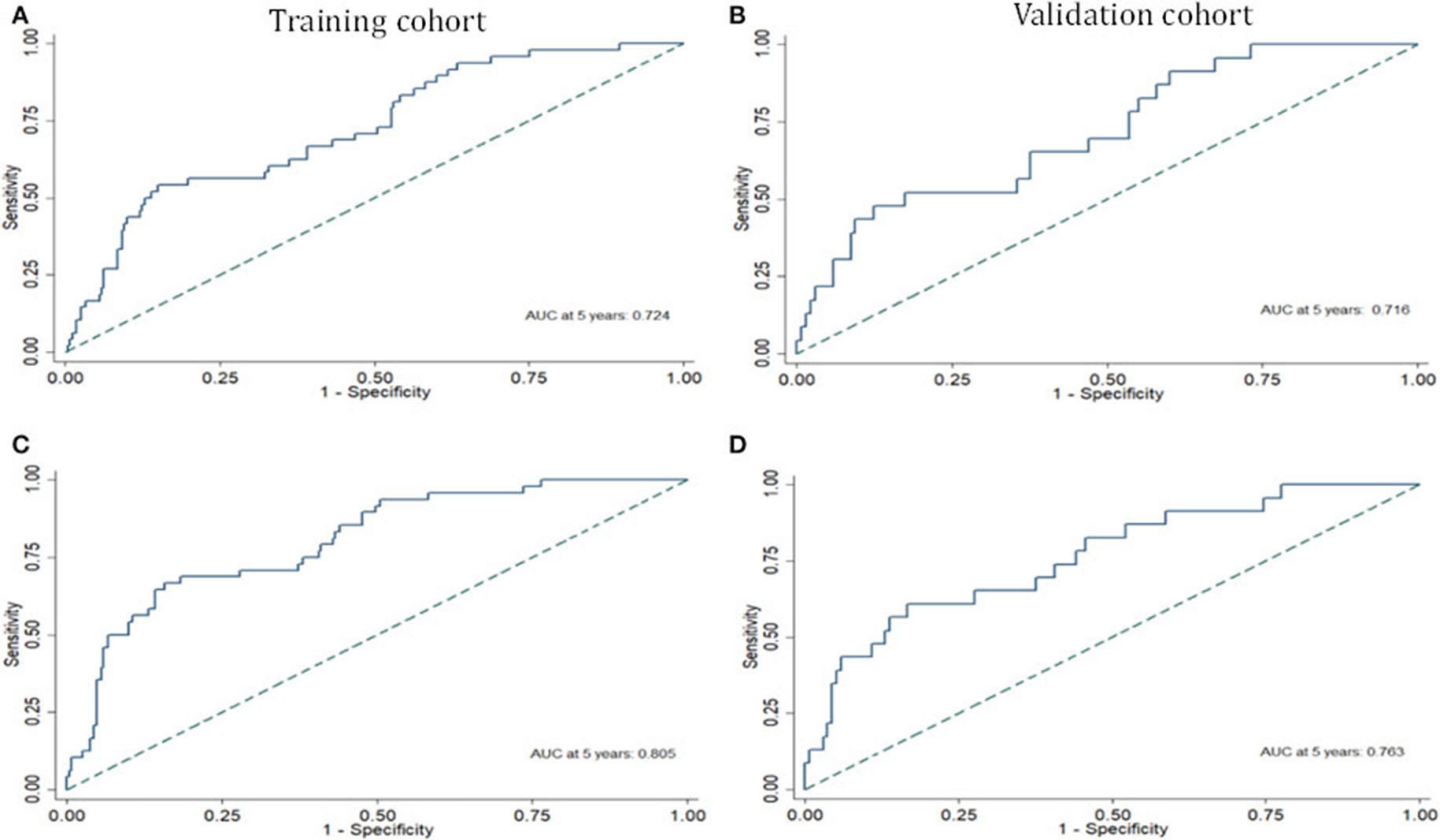
The receiver operating characteristic curve at 5 years based on the six-miRNA-based signature in the training cohort **(A)** and validation cohort **(B)**. The receiver operating characteristic curve at 5 years based on the six-miRNA-based prognostic model in the training cohort **(C)** and validation cohort **(D)**.

**Figure 6:**
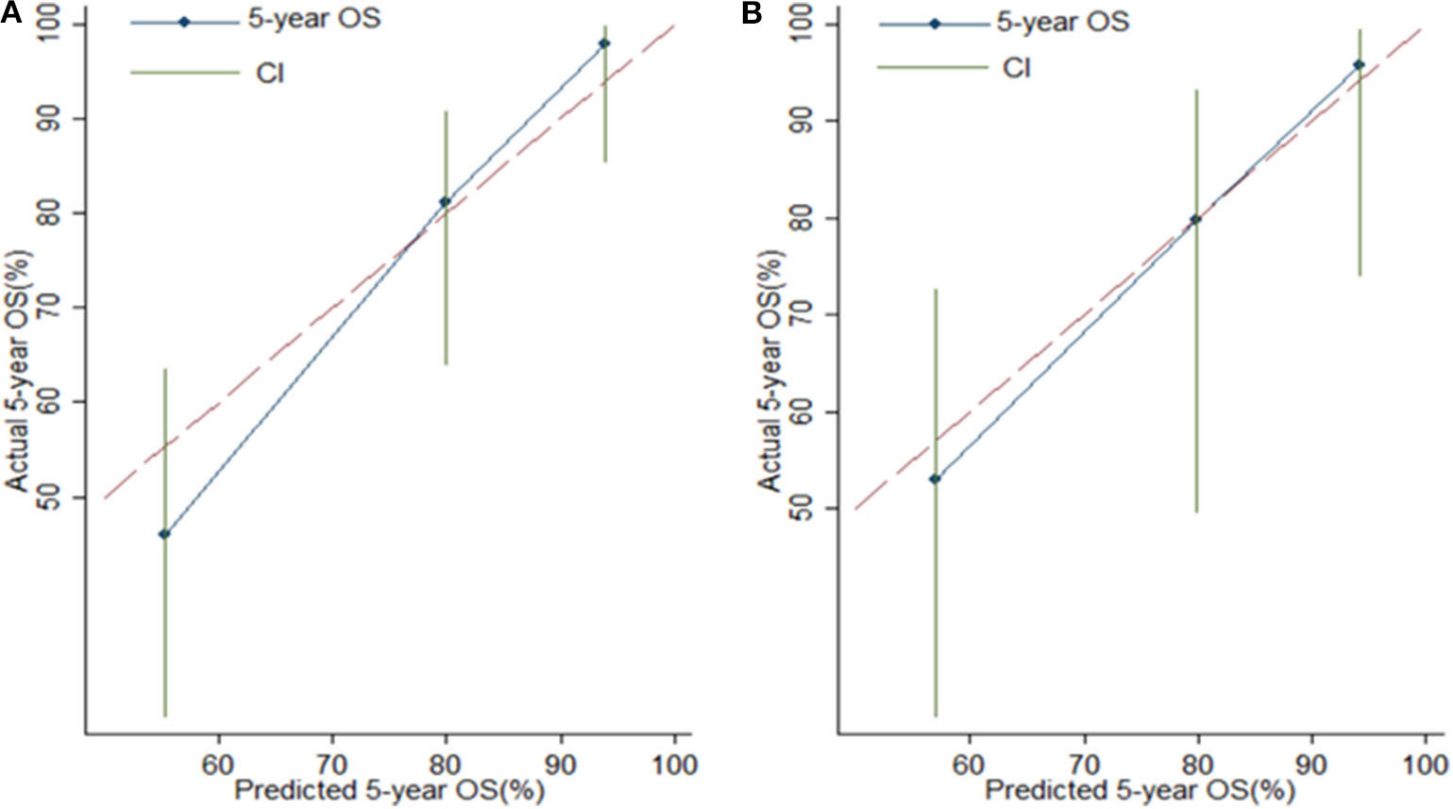
Calibration plots for assessing the agreement between the predicted and the actual overall survival for the six-miRNA-based model. The 45° reference line indicates perfect calibration, where the predicted probabilities are identical to the actual probabilities. Calibration plots of the six-miRNAs-based model in the training cohort **(A)** and validation cohort **(B)**.

**Figure 7:**
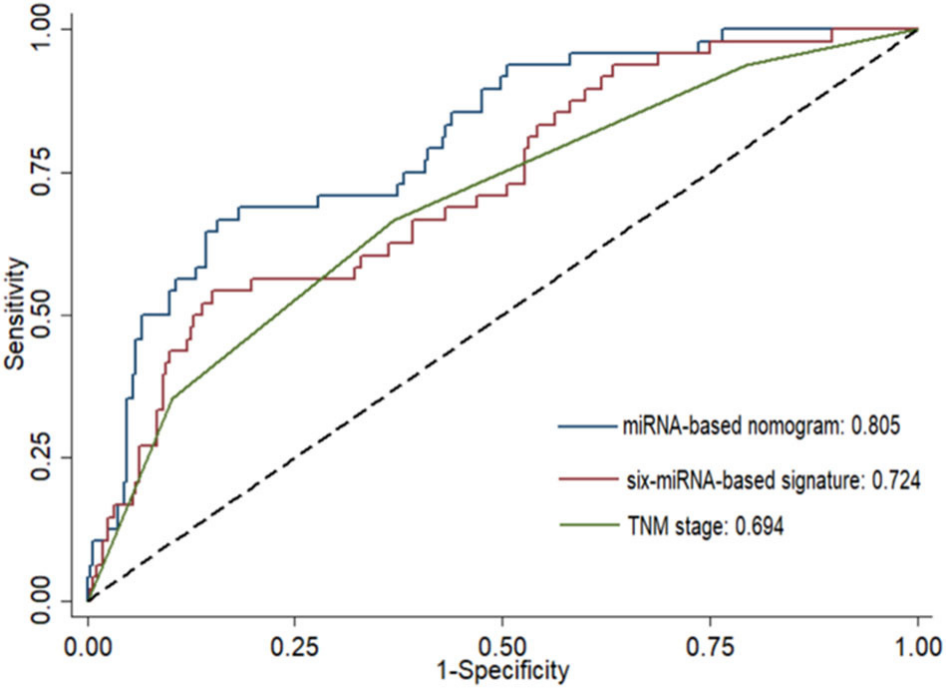
Comparison of the prognostic accuracy at 5 years using the area under receiver operating characteristic curves (AUC) in six-miRNA-based signature, six-miRNA-based prognostic model, and TNM stage.

Risk stratification in patients was performed to assess whether the six-miRNA-based signature could predict OS regardless of TNM stage. The patients with high risk have significantly poorer survival than patients with low risk in TNM stage II (*P* = 0.0027) and TNM stage III (*P* = 0.0130) (Figure 4). Then, to evaluate the discrimination of the six-miRNA-based signature and prognostic nomogram, time-dependent receiver operating characteristic (ROC) curves were used to compare the respective area under the curves (AUC). As a result, AUCs of six-miRNA-based signature were 0.724 and 0.716 in the training cohort and the validation cohort, respectively (Figures 5A,B). Furthermore, we found that there was no significant difference between the T stage, N stage, and risk groups (Table 4). In addition, the AUCs of six-miRNA-based prognostic model were 0.805 and 0.763 in the training cohort and the validation cohort, respectively (Figures 5C,D). Furthermore, the calibration plots showed good agreement between the predicted and actual OS rates in two cohorts when using the new model (Figure 6). These results indicated that the novel six-miRNA-based prognostic model had a favorable discrimination and accuracy prediction for CA patients. Importantly, the AUCs indicated that the six-miRNA-based prognostic model (AUC: 0.805) had better discrimination performance for CA than six-miRNA-based signature (AUC: 0.724) and TNM stage (AUC: 0.694) (Figure 7).

**Table 4:** The correlation between risk groups and clinical features.

	**Low risk**		**High risk**		
**Variables**	***N***	**%**	***N***	**%**	***P***
Age					*P* = 0.773
≤ 69	131	54.36	42	52.50	
>69	110	45.64	38	47.50	
Sex					*P* = 0.496
Male	116	48.13	35	43.75	
Female	125	51.87	45	56.25	
T stage					*P* = 0.595
T1	6	2.49	1	1.25	
T2	47	19.50	11	13.75	
T3	163	67.63	59	73.75	
T4	25	10.37	9	11.25	
N stage					*P* = 0.850
N0	143	59.34	50	62.50	
N1	56	23.24	18	22.50	
N2	42	17.43	12	15.00	
TNM stage					*P* = 0.253
I	48	19.92	11	13.75	
II	92	38.17	37	46.25	
III	70	29.05	18	22.50	
IV	31	12.86	14	17.50	

### MicroRNA-Target Genes Co-expression Network

**Figure 8:**
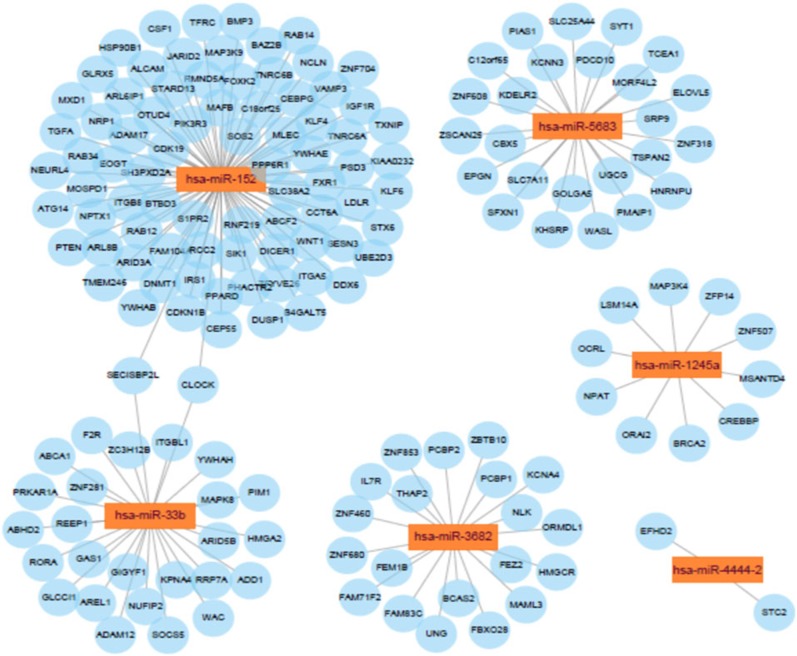
Network for the target genes of the six miRNAs.

Potential connections between microRNAs and target genes were explored by using Cytoscape. As shown in Figure 8, hsa-miR-152 and hsa-miR-33b were the two largest nodes in the network.

### GO and KEGG Pathway Analyses of Predicted Target Genes

**Figure 9:**
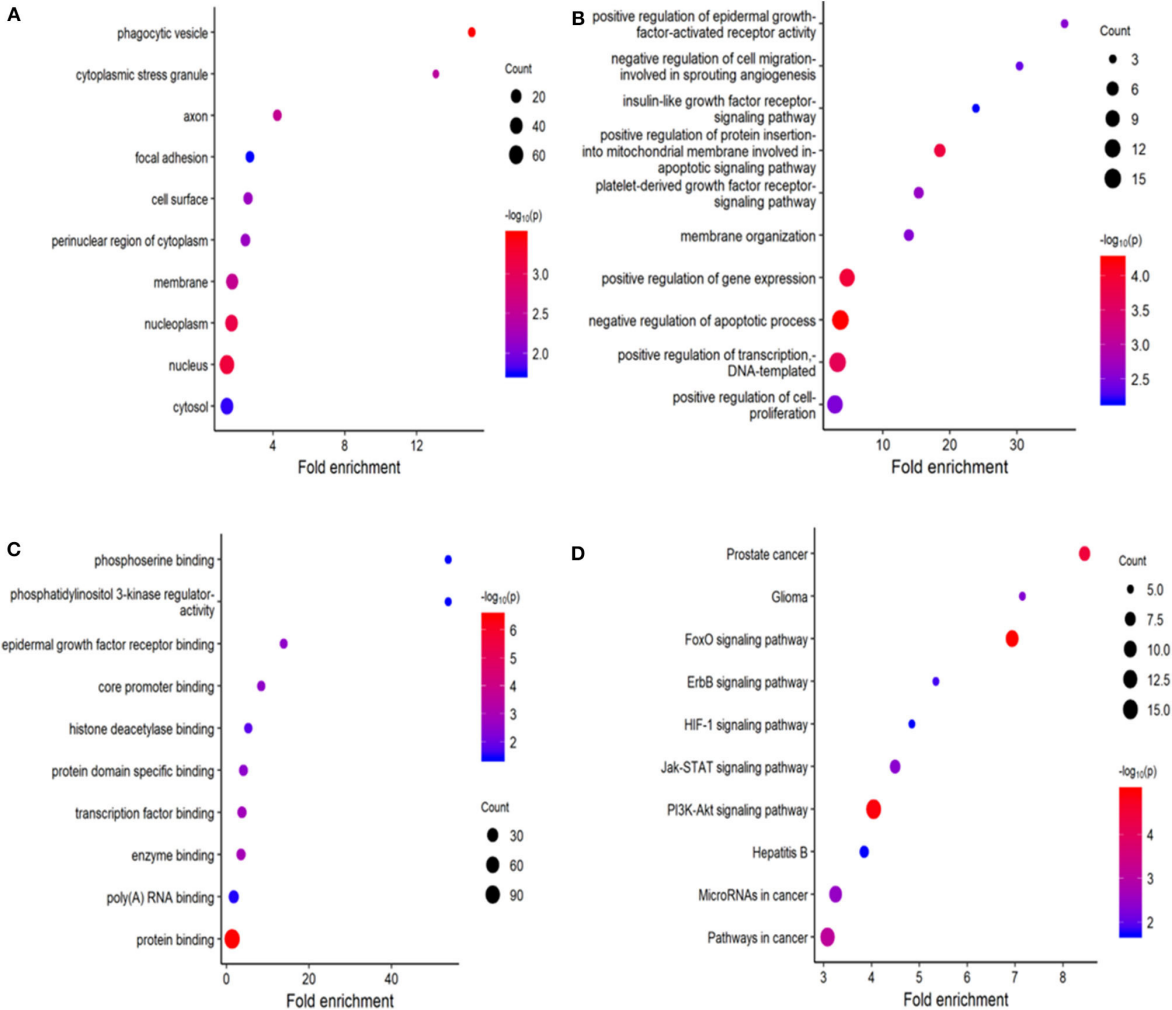
Functional enrichment analysis for predicted target gene of the six miRNAs. **(A–C)** Gene ontology (GO) enrichment analysis: **(A)** cellular component; **(B)** biological process; **(C)** molecular function. **(D)** Kyoto Encyclopedia of Genes and Genomes (KEGG) enrichment analysis. The *x*-axis represents the numbers of genes. The *y*-axis shows the GO terms and KEGG pathway names.

Using miRDB, TargetScan, and miRTarBase, 164 target genes of these six miRNAs were predicted. The GO molecular function (MF) enrichment analysis showed that these genes are dominated by functions of protein binding, poly(A) RNA binding, and enzyme binding (Figure 9A). The GO biological process (BP) enrichment indicated that these genes are mainly involved in regulation of transcript, regulation of apoptotic process, and regulation of gene expression (Figure 9B). The enriched GO cell complement (CC) term of these genes included cytosol, nucleus, and nucleoplasm (Figure 9C). KEGG pathway enrichment analysis indicated that these genes were mainly enriched in PI3K-Akt signaling pathway and FoxO signaling pathway (Figure 9D).

### miRNAs in Risk Score Modulate Biological Processes in CA Cell Line

**Figure 10:**
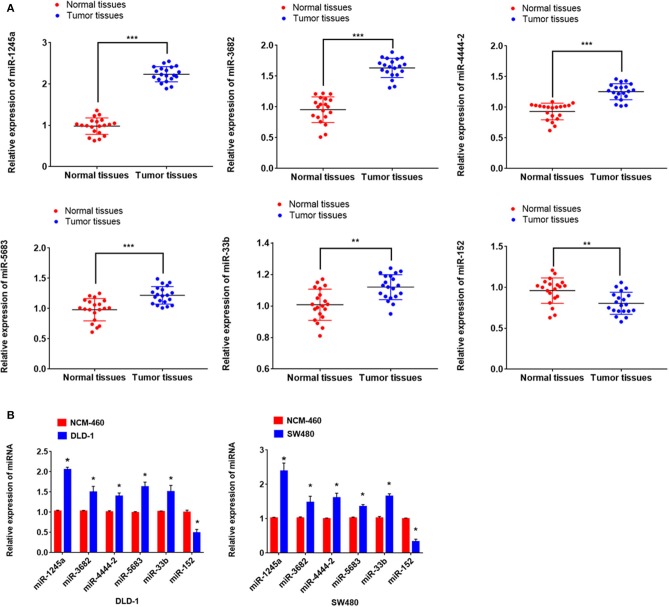
miR-1245a, miR-3682, miR-444-2, miR-5683, and miR-3b were higher in tumor tissues than normal tissues, while miR-152 was low in tumor tissues **(A)**. miR-1245a, miR-3682, miR-444-2, miR-5683, and miR-3b, except miR-152 were significantly high in DLD-1 and SW480 cells normalized to NCM-460 cell **(B)**. *P < 0.05, **P < 0.01, ***P < 0.001 (Student' *t*-test).

**Figure 11:**
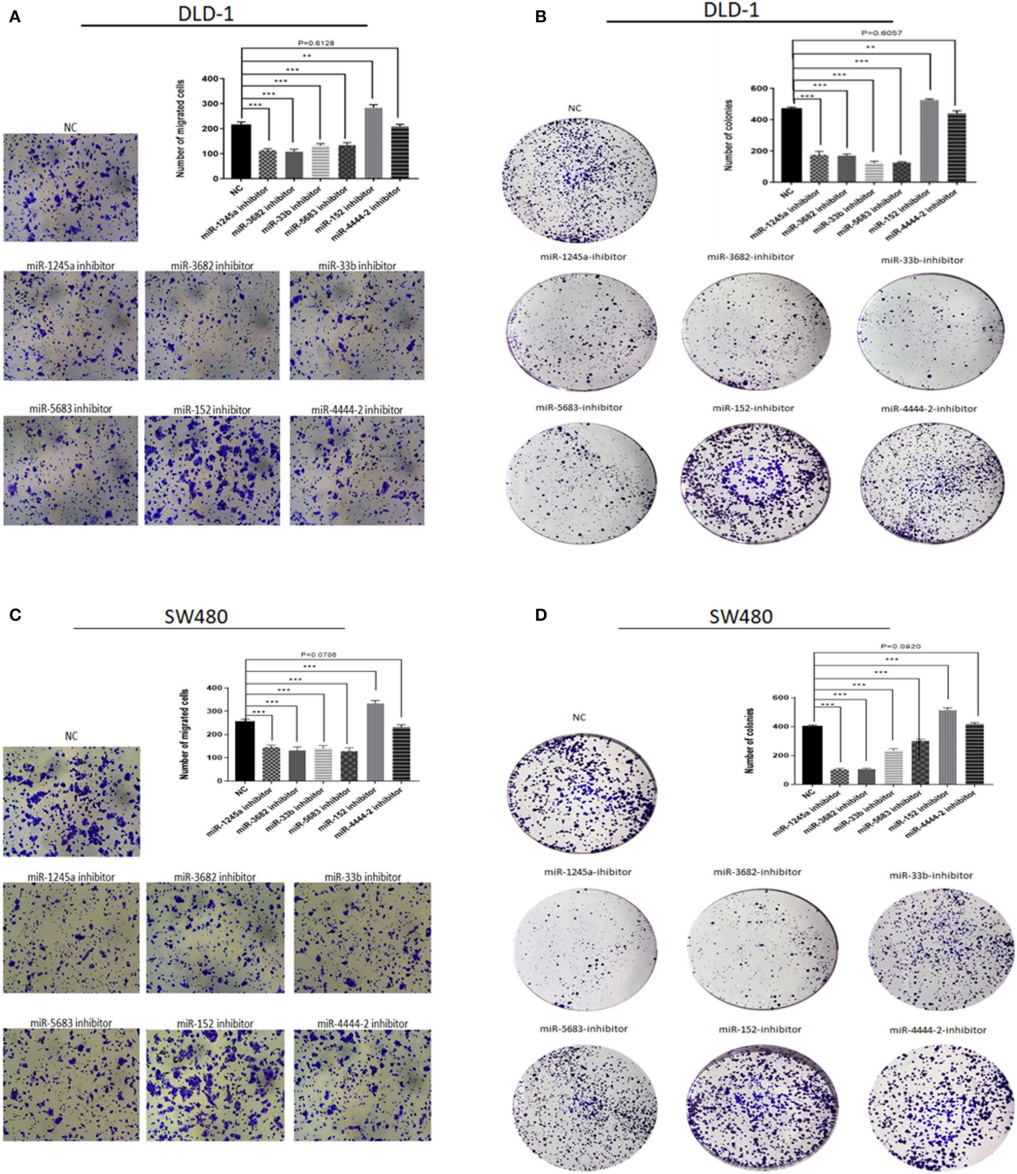
Function experiments of miRNAs in signature. **(A,C)** Representative results of transwell assays of DLD-1 and SW480 cells transfected with miRNA-NC or miRNA-inhibitors. **(B,D)** Representative results of colony formation of DLD-1 and SW480 cells transfected with miRNA-NC or miRNA-inhibitors. **P < 0.01, ***P < 0.001 (Student' *t*-test).

First, we evaluated the expressions of the six miRNAs in 20 pairs in tissues and found that miR-1245a, miR-3682, miR-444-2, miR-5683, and miR-3b were higher in tumor tissues than normal tissues, while miR-152 was low in tumor tissues (Figure 10A). Additionally, in DLD-1 and SW480 cells, miR-1245a, miR-3682, miR-444-2, miR-5683, and miR-3b except miR-152 were significantly high in two CA cell lines normalized to NCM-460 cells (Figure 10B). The six miRNAs were evaluated by cell function assessment in CA cell lines (DLD-1 and SW480). After transfection with the miRNA inhibitors, transwell assays and colony assays were performed. Transwell assays showed that knockdown of miR-1245a, miR-3682, miR-33b, and miR-5683 inhibited the migratory abilities of DLD-1 cells and SW480 cells, whereas knockdown of miR-152 had opposite effects. However, the results showed that miR-4444-2 did not influence the cell's migratory ability (Figures 11A,C). In colony assays, we found that, compared to the negative control, down-regulation of miR-1245a, miR-3682, miR-33b, and miR-5683 inhibited the CA cell proliferation, while the colony was promoted by the knockdown of miR-152. Interestingly, miR-4444-2 still had no effects in cell proliferation (Figures 11B,D).

## Discussion

For the therapy of colon carcinoma, there were few reliable and effective prognostic biomarkers, let alone accurate model for predicting clinical outcome. The prediction of OS in these patients may facilitate individualization of the clinical decision-making process, such as the precise selection of patient pollution for treatment of neo-adjuvant chemotherapy. In this study, we aimed to explore the biological value of miRNAs in CA and tried to develop a prediction model using miRNA expression. In brief, we screened 190 DEMs through comparing tumor tissue with normal tissue in TCGA dataset. Finally, six out of them (miR-1245a, miR-3682, miR-4444-2, miR-5683, miR-33b, and miR-152) were screened as prognosis-related miRNAs and were used to develop a risk score signature. Multivariate COX analysis indicated that this risk score signature was an independent prognosis factor. Then, we built a novel model based on six-miRNA-based signature and other clinical characteristics.

Currently, TNM staging system could provide prognostic information and treatment option. The present study indicated that TNM staging system was a strong independent prognostic factor in the multivariable Cox analysis, which was consistent with previous studies (12). Furthermore, in stratification analysis, the prognostic value of six-miRNA-based signature was revealed to be independent of TNM stage for CA. In addition, TNM staging system cannot provide individualized estimations. Therefore, the six-miRNA-based prognostics model was designed as an easy-to-use nomogram for patients and physicians. Compared to the TNM staging system, our new model has better discrimination and accuracy, suggesting that this model might serve as a potential predictive tool for CA patients. With the more accurate prediction of OS, physicians may be able to provide more accurate recommendations to CA patients.

In the previous studies, although several prognostic models have been developed (13–16), a few models associated with miRNAs were reported. Among these models, a previous study has reported a prognostic miRNA model for predicting OS for CA (16). However, compared with this model, our model has several advantages. Firstly, we selected candidate miRNAs in the DEMs that were analyzed by the LIMMA analysis (FDR < 0.05 and |log2FC| ≥ 2) and then six of these miRNAs were screened from the univariate and multivariate COX analysis, rather than screening directly the candidate variables from a total of 1,881 miRNAs though statistics analysis. Secondly, our study used AUCs and calibration plots to evaluate the discrimination and accuracy of the novel model in the training cohort and the validation cohort. Briefly, in our study, the results indicated that our new model has a good predictive ability in two cohorts. Although the patients in the validation cohort came from primary patients, they were assigned randomly as the validation cohort based on a computer-generated allocation sequence as previously described (10), suggesting that the validation cohort was valid in this study. Thirdly, we further used experiment to explore the role of these six miRNAs in the biological processes in CA.

Among these six prognosis-related miRNAs, miR-1245a, miR-33B, and miR-152 were reported in previous studies for multiple cancers. For miR-1245, Yang et al. reported that it could promote proliferation and invasion of lung cancer cells though targeting BRCA2 (17). In addition, several studies have shown that miR-152 could suppress various cancer types (18–20). For example, Feng et al. suggested that miR-152 could inhibit cell growth in prostate cancer progression (21). Interestingly, several studies found that miR-33b was a tumor suppressor (22, 23). However, miR-33b showed its tumorigenesis and was associated with poor survival in our studies. To date, there were no studies focused on miR-33b in CA. Therefore, further studies were needed to explore the biological process of miR-33b in CA. The other three miRNAs have not been reported in previous studies. Our cell assays indicated that both miR-3682 and miR-5683 could promote proliferation and migration of CA cells, implying that these two prognosis-related miRNAs may employ their biological processes in CA. Moreover, our study showed that miR-4444-2 has no effect on CA cell growth. However, STC2, its predicted target gene, has been revealed in published studies about its tumorigenesis in colorectal carcinoma (24–26), which implied that miR-4444-2 may regulate STC2 though a certain mechanism in CA. Although the precancerous conditions of colon carcinoma, such as ulcerative colitis and Crohn's disease, are the risk factors of colon carcinoma, there were no studies reporting that these six miRNAs correlate with precancerous conditions (27, 28).

Several limitations exist in our studies. Firstly, like the previous studies, detail systematic therapy data, such as surgery, chemotherapy, and radiotherapy, were not accessible, and whether incorporating these factors into our model would improve its performance is unknown. Secondly, the sample size in this study was medium. Therefore, larger multicenter validation should be performed to verify this novel model before application in routine clinical practice.

## Conclusions

In conclusion, we developed a novel model based on six-miRNA score signature and clinical features to predict 5-year survival probabilities for CA patients. This model had higher prognostic value than TNM stage in CA patients. Therefore, this model has the potential to facilitate individualized treatment decisions for CA patients.

## Data Availability Statement

Publicly available datasets were analyzed in this study, these can be found in The Cancer Genome Atlas (https://portal.gdc.cancer.gov/).

## Author Contributions

ZR, YR, YL, LZ, NZ, and ZP: conceptualization. ZR, YL, and ZP: methodology. NZ and ZP: software. ZR, NZ, JP, and ZP: formal analysis. ZP and NZ: resources. ZR, ZP, and BZ: writing—original draft preparation.

### Conflict of Interest

The authors declare that the research was conducted in the absence of any commercial or financial relationships that could be construed as a potential conflict of interest.

## References

[1] Cassidy S Syed BA. Colorectal cancer drugs market. Nat Rev Drug Discov. (2017) 16:525–6. 10.1038/nrd.2017.59 28529321

[2] Siegel RL Miller KD. Cancer statistics, 2019. CA Cancer J Clin. (2019) 69:7–34. 10.3322/caac.21551 30620402

[3] Bartel DP. MicroRNAs: genomics, biogenesis, mechanism, and function. Cell. (2004) 116:281–97. 10.1016/S0092-8674(04)00045-5 14744438

[4] Slattery ML Herrick JS Pellatt DF Stevens JR Mullany LE Wolff E. MicroRNA profiles in colorectal carcinomas, adenomas and normal colonic mucosa: variations in miRNA expression and disease progression. Carcinogenesis. (2016) 37:245–61. 10.1093/carcin/bgv249 26740022 PMC4766359

[5] Zhang Y Li M Ding Y Fan Z Zhang J Zhang H. Serum MicroRNA profile in patients with colon adenomas or cancer. BMC Med Genomics. (2017) 10:23. 10.1186/s12920-017-0260-7 28427387 PMC5399348

[6] Hofsli E Sjursen W Prestvik WS Johansen J Rye M Trano G. Identification of serum microRNA profiles in colon cancer. Br J Cancer. (2013) 108:1712–9. 10.1038/bjc.2013.121 23558896 PMC3668463

[7] Luo X Stock C Burwinkel B Brenner H. Identification and evaluation of plasma microRNAs for early detection of colorectal cancer. PLoS ONE. (2013) 8:e62880. 10.1371/journal.pone.0062880 23690963 PMC3653912

[8] Mao Y Fu Z Zhang Y Dong L Zhang Y Zhang Q. A six-microRNA risk score model predicts prognosis in esophageal squamous cell carcinoma. J Cell Physiol. (2019) 234:6810–9. 10.1002/jcp.27429 30387125

[9] Trino S Lamorte D. MicroRNAs as new biomarkers for diagnosis and prognosis, and as potential therapeutic targets in acute myeloid leukemia. Int J Mol Sci. (2018) 19:E460. 10.3390/ijms19020460 29401684 PMC5855682

[10] Lai J Wang H Pan Z Su F. A novel six-microRNA-based model to improve prognosis prediction of breast cancer. Aging. (2019) 11:649–62. 10.18632/aging.101767 30696800 PMC6366967

[11] Shannon P Markiel A Ozier O Baliga NS Wang JT Ramage D. Cytoscape: a software environment for integrated models of biomolecular interaction networks. Genome Res. (2003) 13:2498–504. 10.1101/gr.1239303 14597658 PMC403769

[12] Marrelli D Morgagni P de Manzoni G Coniglio A Marchet A Saragoni L. Prognostic value of the 7th AJCC/UICC TNM classification of noncardia gastric cancer: analysis of a large series from specialized Western centers. Ann Surg. (2012) 255:486–91. 10.1097/SLA.0b013e3182389b1a 22167003

[13] Zheng P Chen Q Li J Jin C Kang L Chen D. Prognostic significance of tumor deposits in patients with stage iii colon cancer: a nomogram study. J Surg Res. (2019) 245:475–82. 10.1016/j.jss.2019.07.099 31446189

[14] Saso K Myoshi N Fujino S Takenaka Y Takahashi Y Nishimura J. Erratum: a novel prognostic prediction model for recurrence in patients with stage II colon cancer after curative resection. Mol Clin Oncol. (2019) 11:213. 10.3892/mco.2019.1879 31316775 PMC6604394

[15] Yu C Zhang Y. Development and validation of a prognostic nomogram for early-onset colon cancer. Biosci Rep. (2019) 39:BSR20181781. 10.1042/BSR20181781 31142625 PMC6617053

[16] Chen F Li Z Zhou H. Identification of prognostic miRNA biomarkers for predicting overall survival of colon adenocarcinoma and bioinformatics analysis: a study based on The Cancer Genome Atlas database. J Cell Biochem. (2019) 120:9839–49. 10.1002/jcb.28264 30536901

[17] Yang L Wang J Fan Y Yu K Jiao B Su X. Hsa_circ_0046264 up-regulated BRCA2 to suppress lung cancer through targeting hsa-miR-1245. Respir Res. (2018) 19:115. 10.1186/s12931-018-0819-7 29891014 PMC5996480

[18] Chen K Zhang L. LINC00339 regulates ROCK1 by miR-152 to promote cell proliferation and migration in hepatocellular carcinoma. J Cell Biochem. (2019) 120:14431–43. 10.1002/jcb.28701 31081143

[19] You W Zhang X Ji M Yu Y Chen C Xiong Y. MiR-152-5p as a microRNA passenger strand special functions in human gastric cancer cells. Int J Biol Sci. (2018) 14:644–53. 10.7150/ijbs.25272 29904279 PMC6001653

[20] Lu ZW Du MY Qian LX Zhang N Gu JJ Ding K. MiR-152 functioning as a tumor suppressor that interacts with DNMT1 in nasopharyngeal carcinoma. Onco Targets Ther. (2018) 11:1733–41. 10.2147/OTT.S154464 29628766 PMC5877490

[21] Feng F Liu H Chen A. miR-148-3p and miR-152-3p synergistically regulate prostate cancer progression via repressing KLF4. J Cell Biochem. (2019) 120:17228–39. 10.1002/jcb.28984 31104329

[22] Zhai S Zhao L Lin T Wang W. Downregulation of miR-33b promotes non-small cell lung cancer cell growth through reprogramming glucose metabolism miR-33b regulates non-small cell lung cancer cell growth. J Cell Biochem. (2019) 120:6651–60. 10.1002/jcb.27961 30368888 PMC6587718

[23] Zhao M Qi M Li X Hu J Zhang J Jiao M. CUL4B/miR-33b/C-MYC axis promotes prostate cancer progression. The Prostate. (2019) 79:480–8. 10.1002/pros.23754 30609075

[24] Zhang C Chen S Ma X Yang Q Su F Shu X. Upregulation of STC2 in colorectal cancer and its clinicopathological significance. Onco Targets Ther. (2019) 12:1249–58. 10.2147/OTT.S191609 30863092 PMC6389002

[25] Li Q Zhou X Fang Z Pan Z. Effect of STC2 gene silencing on colorectal cancer cells. Mol Med Rep. (2019) 20:977–84. 10.3892/mmr.2019.10332 31173256 PMC6625197

[26] Chen B Zeng X He Y Wang X Liang Z Liu J. STC2 promotes the epithelial-mesenchymal transition of colorectal cancer cells through AKT-ERK signaling pathways. Oncotarget. (2016) 7:71400–16. 10.18632/oncotarget.12147 27662663 PMC5342087

[27] Yang L Bian Y Li Z Yan Y Li J Li W. Identification of potential biomarkers and pathways in ulcerative colitis with combined public mRNA and miRNA expression microarray data analysis. J Gastrointest Oncol. (2019) 10:847–58. 10.21037/jgo.2019.06.06 31602322 PMC6776810

[28] Zheng Y Ge W Ma Y Xie G Wang W Han L. miR-155 Regulates IL-10-producing CD24(hi)CD27(+) B cells and impairs their function in patients with Crohn's disease. Front Immunol. (2017) 8:914. 10.3389/fimmu.2017.00914 28824639 PMC5540954

